# miR-199-5p regulates spermiogenesis at the posttranscriptional level via targeting *Tekt*1 in allotriploid crucian carp

**DOI:** 10.1186/s40104-022-00693-4

**Published:** 2022-04-13

**Authors:** Shengnan Li, Qiubei Wang, Lu Huang, Siyu Fan, Ting Li, Yuqing Shu, Chun Zhang, Yi Zhou, Qingfeng Liu, Kaikun Luo, Min Tao, Shaojun Liu

**Affiliations:** 1 State Key Laboratory of Developmental Biology of Freshwater Fish, College of Life Sciences, Hunan Normal University, Changsha, 410081 PR China; 2 Guangdong Laboratory for Lingnan Modern Agriculture, Guangzhou, 510642 PR China

**Keywords:** Allotriploid crucian carp, miR-199-5p, Sperm abnormalities, Sperm flagella, Spermiogenesis, *Tekt*1

## Abstract

**Background:**

Sperm abnormalities are one of the primary factors leading to male sterility, but their pathogenesis is still unclear. Although miRNAs are suggested to exert important roles in the regulation of spermatogenesis at both transcriptional and posttranscriptional levels, little is currently known regarding the regulation of sperm flagella assembly by microRNAs (miRNAs). The role of miRNAs in the development of sperm abnormalities in sterile triploid fish has not been studied.

**Results:**

In this study, we found that miR-199-5p was widely expressed in all detected tissues of different-ploidy crucian carp. As one of the testis-specific candidate markers, *Tekt*1 was predominantly expressed in the testis. Quantitative real-time PCR (qRT-PCR) analyses showed that the expression trend of miR-199-5p was exactly opposite to that of *Tekt*1. Through bioinformatics analysis, we identified a putative miR-199-5p binding site in the *Tekt*1 mRNA. We further identified *Tekt*1 as a target of miR-199-5p using luciferase reporter assay. Finally, we confirmed that miR-199-5p was necessary for sperm flagellar assembly and spermatogenesis in vivo via intraperitoneal injection of miR-199-5p antagomir or agomir in diploid red crucian carp. Moreover, miR-199-5p gain-of-function could lead to spermatids apoptosis and abnormal spermatozoa structure, which is similar to that of allotriploid crucian carp.

**Conclusions:**

Our studies suggested that abnormally elevated miR-199-5p inhibited the sperm flagella formation in spermiogenesis by negatively regulating the expression of *Tekt*1, thereby causing sperm abnormalities of male allotriploid crucian carp.

## Introduction

Red crucian carp (*Carassius auratus* red var.) and common carp (*Cyprinus carpio* L.) are members of Cyprinidae. In previous studies, Bisexual fertile allotetraploid hybrids were produced by the intergeneric hybridization of female red crucian carp and male common carp. Subsequently, the allotetraploid hybrid lineage was generated by continuous self-crossing [1–3]. Allotriploid crucian carp was obtained from the hybridization of female red crucian carp and male allotetraploid hybrids, which was sterile and owned many merits, such as faster growth rate, stronger disease resistance and stress resistance, and high-quality meat [4–6]. An earlier study demonstrated that allotriploids can only produce watery semen during the spermiation period but cannot produce normal semen with milky white. Instead, the germ cells developed into round spermatids and then degenerated [7]. Changes during meiosis and spermiogenesis result in the deficiency in normal spermatozoa [8]. Moreover, the transmission electron microscopy of allotriploid testicular sections revealed that, during spermiogenesis, the elongated spermatids failed to form integrated tails and normal nuclei [9]. Thus, a better understanding of the regulatory mechanisms of sterility in male triploid fish will provide important information for both reproductive endocrinologists and fish breeding experts.

Spermatogenesis is a complex and highly regulated process, in which diploid primordial germ cells proliferate and differentiate to form mature haploid spermatozoa for sexual reproduction [10]. Spermiogenesis, the last stage of spermatogenesis, is characterized by complex morphological changes through which round spermatids differentiate into elongated spermatids and eventually into motile spermatozoa [11, 12]. A series of morphologic changes occurs during this stage, including formation and development of the flagellum and acrosome, nuclear condensation and elongation, reorganization of organelles and removal of cytoplasm during spermiation [13, 14]. In cyprinids, spermiogenesis can be divided into four stages [15], and the orderly flagella formation is a key stage of normal spermatozoa formation.

Tektins (TEKTs) are constitutive filamentous proteins related to microtubules in cilia, flagella, basal bodies, and centrioles [16, 17], and are preferentially expressed in the spermatids that form the tail in the testis [18]. They were originally found in sea urchins sperm tails (Tektin A, B, and C) [19], and were highly coiled-coil molecules that form filaments in the wall of ciliary and flagellar microtubules [20, 21]. In mammals, the TEKT family consists of five members (Tektin 1–5), which have been identified in testis and spermatozoa [22, 23]. They are mainly involved in the formation of the sperm flagellar axoneme and its accessory structures, which together play an important role in sperm motility. Tekt1 is a member of TEKT family, which is associated with sperm flagella formation, and may be correlated with flagellar stability and sperm motility [24]. In a previous study, *Tekt*1 mRNA was initially found to be localized in spermatocytes and round spermatids in mouse testis and was deemed to be involved in spermatogenesis [25]. Subsequently, Oiki et al. found that *Tekt*1 mRNA existed in sperm flagella and apical regions of the acrosome cap in spermatozoa of mouse, bull, and rat [22]. Besides, *Tekt*1 mutation or defect was an important cause of immobility or asthenozoospermia in humans, dogs, and other species [26–29]. Our previous study demonstrated that *Tekt*1, as one of the testis-specific candidate markers for sterility, was expressed at a higher level in the testis of fertile diploid fish than in that of sterile allotriploid fish, indicating that low *Tekt*1 expression may inhibit the maturation of spermatids into motile sperm in sterile allotriploid fish [9].

MicroRNAs (miRNAs) are a novel class of short endogenous single-stranded non-coding RNAs with a length of approximately 22 nt [30, 31]. Just like mRNAs, miRNAs have attracted widespread attention because it is clear that they regulate genes involved in many important processes, including spermatogenesis [32–35]. Previous studies of miRNAs mainly focus on spermatogonial stem cells, spermatocyte meiosis, spermiogenesis in mammals [36, 37]. However, to the best of our knowledge, little is known about the functions and molecular mechanisms of miRNAs and miRNA-mediated regulation of spermiogenesis in teleost fish during sexual maturation.

The current research on miR-199-5p is related to the occurrence of cancer. For instance, overexpression of miR-199-5p inhibited the growth and metastasis of colorectal cancer cells through downregulation of ROCK1 expression [38]. miR-199-5p overexpression suppressed tumor growth in papillary thyroid cancer in vivo by downregulating SNAI1 [39]. However, the functions of miR-199-5p and its regulatory mechanism for target genes have not been identified in fish, and the role of miR-199-5p in spermiogenesis has not been reported. Our previous study revealed that miR-199-5p in the testis of allotriploid fish might act on the *Tekt*1 3′-UTR, resulting in the *Tekt*1 downregulation [40].

In the current study, the interaction of miR-199-5p and *Tekt*1 was verified using a dual-luciferase reporter system. Besides, *Tekt*1 mRNA and miR-199-5p were detected in the testis of different-ploidy crucian carp from the pre-spermiation and spermiation periods, and gain-of-function and loss-of-function experiments were performed to explore the effects of *Tekt*1 on spermiogenesis. Our study unequivocally suggested that miR-199-5p might play a significant role in sperm flagella formation by posttranscriptionally silencing *Tekt*1 in allotriploid crucian carp, providing evidence to enrich our understanding of the regulation of spermiogenesis in fish.

## Materials and methods

### Ethics statement

All animal care procedures were performed pursuant to experimental protocols approved by the Animal Care Committee of Hunan Normal University (authorization number: 2018–191, 15 March 2018). The Administration of Affairs Concerning Animal Experimentation Guidelines stated that was acquired approval from the Science and Technology Bureau of China.

### Experimental animals collection and sample preparation

Diploid red crucian carp and allotetraploid hybrids were collected from the State Key Laboratory of Developmental Biology of Freshwater Fish at Hunan Normal University. During reproductive season in April 2019, 10 mature male diploid red crucian carp and 10 mature male allotetraploid hybrids were selected as the paternal parents, and 20 mature female diploid red crucian carp were chosen as the maternal parents, respectively. Following artificial insemination, diploid red crucian carp were produced by self-crossing of diploid red crucian carp, and allotriploid crucian carp were generated by fertilization of diploid red crucian carp eggs and allotetraploid hybrids sperm. The embryos developed in the culture dishes at the water temperature of 21–22 °C. Fish were cultivated in open pools (0.067 ha) with suitable pH (7.0–8.5), water temperature (22–24 °C), dissolved oxygen content (5.0–8.0 mg/L), and adequate forage. A total of nine diploid red crucian carp and nine allotriploid crucian carp (six males and three females, respectively) were randomly collected during spermiation and pre-spermiation periods (April and November). The ploidy of all individual fish was confirmed by flow cytometer (Cell Counter Analyzer, Partec, Germany) as described by Liu et al. [41]. The individuals were euthanized with 100 mg/L MS-222 (Sigma-Aldrich, St. Louis, MO, USA). Subsequently, the telencephala, hypothalami, pituitaries, testes, kidneys, livers, hearts, spleens, skins and muscles were excised surgically from the male fish and only the ovaries were excised surgically from the female fish, snap-frozen in liquid nitrogen, and then stored at − 80 °C for the tissue expression analysis of *Tekt*1 gene and miR-199-5p.

### RNA extraction and quantitative real-time PCR (qRT-PCR) analysis

Total RNA was extracted using the Invitrogen TRIzol® Reagent (Invitrogen, Carlsbad, CA, USA) following the manufacturer’s protocols. The integrity and concentration of the RNA samples were detected by electrophoresis on a 1.2% denaturing agarose gel and measured by OD260/OD280 using a spectrophotometer, respectively. Total RNA with good results was immediately utilized for further experiments.

For mRNA quantification, reverse transcription was conducted using the PrimeScript™ RT reagent Kit with gDNA Eraser (Perfect Real Time) (Takara, Tokyo, Japan) according to the manufacturer’s instructions. The qRT-PCR was conducted on a Prism 7500 Sequence Detection System (Applied Biosystems, Foster City, CA, USA) and in a volume of 10 μL containing 5 μL PowerUp™ SYBR™ Green Master Mix (Applied Biosystems, Foster City, CA, USA), 1 μL of equally mixed cDNA, 0.5 μL of each primer, and 3 μL ddH_2_O. The qRT-PCR procedure was 50 °C for 2 min, 95 °C for 10 min, followed by 40 cycles at 95 °C for 15 s and 60 °C for 1 min. *Tekt*1 was analyzed using the specific primers. The *β*-*actin* (a housekeeping gene) was used as the internal control.

Likewise, the miRNA expression analysis was carried out using the same total RNA used for mRNA quantification. For miRNA quantification, reverse transcription was carried out using the miScript® II RT Kit (Qiagen, Valencia, CA, USA) according to the manufacturer’s handbook. The qRT-PCR was carried out on the above instrument using the miScript® SYBR® Green PCR Kit (Qiagen, Valencia, CA, USA). The 10 μL reaction solution included 1 μL of equally mixed cDNA, 5 μL of 2× QuantiTect® SYBR Green PCR Master Mix, 0.5 μL of 10× miScript Universal Primer, 0.5 μL of specific forward primer, and 3 μL of RNase-free water. The qRT-PCR procedure was 95 °C for 15 min, followed by 40 cycles at 94 °C for 15 s, 52 °C for 35 s, and 70 °C for 40 s. miR-199-5p was analyzed using the specific forward primers. The U6 small nuclear RNA (a housekeeping miRNA) was used as the internal control.

To ensure the accuracy of qRT-PCR results, the analysis of each sample was repeated four times. Each sample was assessed in quadruplicate, and relative gene or miRNA expression was calculated using the 2^-ΔΔCt^ method after normalization to the level of *β*-*actin* or U6, respectively [42]. All primers for qRT-PCR were listed in Table 1.

**Table 1:** Primers used for qRT-PCR

Primer name	Primer sequence (from 5′ to 3′)	Usage
*Tekt*1-QS	CTGACTGAGAGACAGAAGCGGG	*Tekt*1 qRT-PCR
*Tekt*1-QA	ATTTCACTGAACGAAGGAGCCT	*Tekt*1 qRT-PCR
miR-199-5p	CCCAGTGTTCAGACTACCTGTTC	miRNA qRT-PCR
*β-actin*-S	TCCCTTGCTCCTTCCACCA	Internal control
*β-actin*-A	GGAAGGGCCAGACTCATCGTA	Internal control
U6-F	CGCTTCGGCAGCACATATAC	Internal control
U6-R	TTCACGAATTTGCGTGTCA	Internal control

### Agomir and antagomir

miR-199-5p agomir, miR-199-5p antagomir and their corresponding negative controls (agomir NC and antagomir NC) were chemically synthesized by RiboBio (RiboBio, Guangzhou, China). They were dissolved in phosphate-buffered saline (PBS) before injection. Fifteen male diploid red crucian carp weighing approximately 25 g and three male allotriploid crucian carp were randomly selected from the State Key Laboratory of Developmental Biology of Freshwater Fish at Hunan Normal University during pre-spermiation period (December). These fish for each treatment were separately cultured in a pool with suitable illumination, water temperature, dissolved oxygen content, and adequate forage, and three replicates were set for this experiment. Diploids received a 0.25 mL intraperitoneal injection of miR-199-5p antagomir at a dose of 60 mg/kg body weight once a week (the first day) or miR-199-5p agomir at a dose of 20 mg/kg body weight twice a week (the first day and fourth day). An equal amount of antagomir NC or agomir NC was injected into the other fish. The untreated groups and allotriploids were taken as control. The semen was obtained by gently squeezing the belly of diploid red crucian carp. Subsequently, the testis sample was gently dissected from each diploid and allotriploid fish after one week, respectively. Each testis sample was divided into four portions for gene expression analysis, protein expression analysis, histological analysis and electron microscope analysis.

### Target validation of luciferase reporter assay

RNAhybrid (https://bibiserv.cebitec.uni-bielefeld.de/rnahybrid/), PITA (https://genie.weizmann.ac.il/pubs/mir07/mir07_dyn_data.html) and miRanda (http://www.bioinformatics.com.cn/local_miranda_miRNA_target_prediction_120) were used to predict whether miR-199-5p targeted *Tekt*1. The site of miR-199-5p binding with *Tekt*1 was predicted by the RNA hybrid. To generate the 3′-UTR luciferase reporter construct, the full length of the 3′-UTR sequence of *Tekt*1 containing the predicted miR-199-5p binding site obtained from diploid red crucian carp was cloned by standard procedures into the downstream of the stop codon of the firefly luciferase gene in the pMIR-REPORT Luciferase vector (Ambion, Austin, TX, USA) using PCR generated fragment to generate *Tekt*1–3′-UTR-wt. The miR-199-5p binding site in the 3′-UTR sequence of *Tekt*1 was deleted, amplified via overlap PCR and cloned into the pMIR-REPORT Luciferase vector to generate *Tekt*1–3′-UTR-mutant. HEK293T cells were obtained from the Cell Bank of the Chinese Academy of Sciences (Shanghai, China) and were cultivated in dishes for 24 h with Dulbecco’s modified Eagle’s medium (DMEM) (Gibco, Gaithersburg, MD, USA) containing 10% fetal bovine serum (FBS) (Gibco, Gaithersburg, MD, USA) and 1% penicillin/streptomycin (100 IU/mL; 100 μg/mL) (Invitrogen, Carlsbad, CA, USA) at 37 °C in a humidified incubator with 5% CO_2_ and 95% air (Thermo Scientific, Wilmington, DE, USA) prior to transfection.

For luciferase reporter assay, HEK293T cells cultured in 96-well plates (Corning Inc., NY, USA) were maintained in DMEM with 10% FBS. miR-199-5p mimic and its corresponding negative control (mimic NC) used in this study were chemically synthesized by OBiO (OBiO, Shanghai, China). To identify whether miR-199-5p targeted *Tekt*1, the cells were co-transfected with either wild-type or mutant *Tekt*1 3′-UTR constructs with or without a final concentration of 100 nmol/L miR-199-5p mimic or mimic NC in the serum-free medium using Lipofectamine™ 2000 Transfection Reagent (Invitrogen, Carlsbad, CA, USA) following the manufacturer’s protocols. At 48 h after transfection, the cells were washed with PBS three times and lysed with passive lysis buffer (PLB) (Promega, Maddison, WI, USA). Subsequently, the firefly luciferase and *Renilla* luciferase activities were measured using the Dual-Luciferase Reporter Assay System (Promega, Maddison, WI, USA) according to the manufacturer’s instructions. Each sample was measured after adding firefly luciferase substrate and adding *Renilla* substrate. *Renilla* luciferase activity served as an internal control to normalize firefly luciferase activity. Six parallel groups were set up in each experiment to eliminate the accidental errors introduced in the operation as much as possible.

### Western blot analysis

Total protein was isolated using Tissue or Cell Total Protein Extraction Kit (Sangon Biotech, Shanghai, China) following the manufacturer’s instructions after the testis was washed with Tris-buffered saline (TBS) three times. Protein concentration was measured using the Modified Bradford Protein Assay Kit (Sangon Biotech, Shanghai, China) pursuant to the manufacturer’s recommendations as previously described. Total protein was denatured after boiling for 10 min and resolved on 10% sodium dodecyl sulphate-polyacrylamide gel electrophoresis (SDS-PAGE) gels. After that, the resolved protein was electrotransferred to methanol-activated polyvinylidene difluoride (PVDF) transfer membranes (Thermo Scientific, Wilmington, DE, USA). Subsequently, the membranes were blocked with 5% nonfat milk in TBS for 1 h and incubated with the primary antibodies overnight at 4 °C. The membranes were then washed with TBS solution supplemented with Tween-20 (TBST) three times (10 min/time), and incubated with the secondary antibodies for 1 h at room temperature. Specific proteins were detected with Alkaline Phosphatase-conjugated goat anti-rabbit (1:1500; Proteintech, Rosemont, IL, USA, Cat# SA00002–2) or Alkaline Phosphatase-conjugated Affinipure goat anti-mouse antibodies (1:1500; Proteintech, Rosemont, IL, USA, Cat# SA00002–1) and the BCIP/NBT Alkaline Phosphatase Color Development Kit (Sigma-Aldrich, St. Louis, MO, USA) according to the manufacturer’s handbook. Antibody binding to Tekt1 (1:5000; rabbit polyclonal, Genscript, Nanjing, China) was obtained by immunizing rabbits using the expressed production (all amino acids of Tekt1 of diploid red crucian carp) as the specific antigen. Antibody binding to β-actin (1:3000; mouse monoclonal, Proteintech, Rosemont, IL, USA, Cat# 66009–1-Ig) was commercially purchased.

### Hematoxylin and eosin (H&E) staining

One week after the first intraperitoneal injection of miR-199-5p antagomir or agomir, the testis was collected and fixed in Bouin’s fixative (Phygene, Fuzhou, China) overnight, dehydrated in an increasing ethanol gradient, cleared in xylene, embedded in paraffin, sectioned into 5 μm slices using a Leica RM2016 rotary microtome (Leica Biosystems GmbH, Nussloch, Germany), dewaxed with xylene, rehydrated with ethanol, and stained with hematoxylin and eosin (H&E) (Solarbio, Beijing, China), as described everywhere. The testis sections were examined and photographed using an Olympus microscope CX41 (Olympus Co., Tokyo, Japan).

### Electron microscope analysis of spermatozoa

One week after the first intraperitoneal injection of miR-199-5p antagomir or agomir, the semen was collected and diluted with Hank’s Balanced Salt Solution (HBSS). Then, the diluted semen was transferred into 2.5% glutaraldehyde solution (Ted Pella Inc., Redding, CA, USA). The semen was added dropwise onto coverslips, dehydrated in an increasing ethanol gradient, and air-dried. Likewise, the testis was withdrawn and minced in HBSS to allow the spermatozoa to exit, and the residue was eventually filtered out. The cell suspensions were fixed in 2.5% glutaraldehyde solution, added dropwise onto coverslips, dehydrated in an increasing ethanol gradient, and air-dried. Finally, they were subjected to atomized gilding and were observed with a JSM-6360LV scanning electron microscope (SEM) (JEOL, Tokyo, Japan), as described everywhere.

### Data analysis

All data were expressed as mean ± S.E.M. unless otherwise stated. The statistical analysis was performed using SPSS 19.0 software (IBM, Chicago, IL, USA) to detect a significant difference with one-way analysis of variance (ANOVA) followed by LSD and Duncan post-hoc tests. To evaluate the luciferase reporter assay, unpaired Student’s *t*-tests were performed. Differences between means were considered significant at *P < 0.05, **P < 0.01, or ***P < 0.001. Graphics were drawn using GraphPad Prism 7 (GraphPad Software, San Diego, CA, USA).

## Results

### Tissue distribution of *Tekt*1 mRNA and miR-199-5p by qRT-PCR

**Fig. 1:**
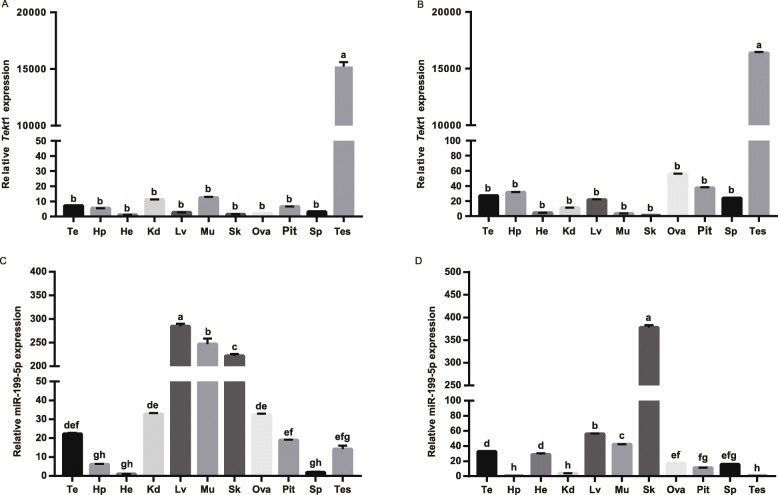
Expression profiles of *Tekt*1 and miR-199-5p in different tissues of different-ploidy crucian carp. **A** The tissue distribution of *Tekt*1 mRNA in diploids. **B** The tissue distribution of *Tekt*1 mRNA in allotriploids. **C** The tissue distribution of miR-199-5p in diploids. **D** The tissue distribution of miR-199-5p in allotriploids. Tc, telencephalon; Hp, hypothalamus; He, heart; Kd, kidney; Lv, liver; Mu, muscle; Sk, skin; Ova, ovary; Pit, pituitary; Sp, spleen; Tes, testis. Data were presented as mean ± S.E.M. (*n* = 3). The values with different lowercase letters differed significantly in expression level (*P* < 0.05)

The expressions of *Tekt*1 mRNA and miR-199-5p in various tissues from the two different-ploidy crucian carp during the spermiation period, as determined by qRT-PCR, were shown in Fig. 1. *Tekt*1 mRNA was widely expressed in all tested tissues in diploids and allotriploids, but especially highly expressed in the testis (Fig. 1A, B). miR-199-5p was widely expressed in all tested tissues in diploids and allotriploids. The expression of miR-199-5p was higher in the liver, muscle and skin of diploids, but lower in the testis, ovary, kidney, pituitary, telencephalon, hypothalamus, spleen and heart (Fig. 1C). However, different from diploids, miR-199-5p was mainly expressed in the skin of allotriploids, and expressed at lower levels in the telencephalon, hypothalamus, heart, kidney, liver, muscle, ovary, pituitary, spleen and testis (Fig. 1D).

### Comparative expression of *Tekt*1 mRNA and miR-199-5p in different-ploidy crucian carp

**Fig. 2:**
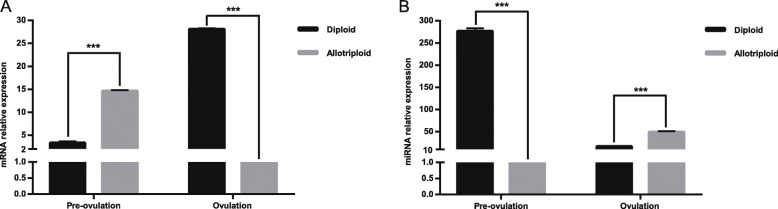
Expression profile in the testis of different-ploidy crucian carp during the pre-spermiation and spermiation periods. **A** The relative expression of *Tekt*1 in the testis of different-ploidy fish. **B** The relative expression of miR-199-5p in the testis of different-ploidy fish. Data were presented as mean ± S.E.M. (*n* = 3). Asterisk (*) indicated significant difference (***P < 0.001)

The expression patterns of *Tekt*1 and miR-199-5p in the testis of the different-ploidy crucian carp during the pre-spermiation and spermiation periods were assessed by qRT-PCR. As shown in Fig. 2, the expressions of *Tekt*1 and miR-199-5p differed greatly among the two different-ploidy fish. In the pre-spermiation period, allotriploids showed a high-level expression of *Tekt*1 in the testis, but a low-level expression of miR-199-5p compared with diploids. In the spermiation period, allotriploids showed a low-level expression of *Tekt*1 in the testis, but a high-level expression of miR-199-5p compared with diploids.

### miR-199-5p acts directly at the 3′-UTR of *Tekt*1

**Fig. 3:**
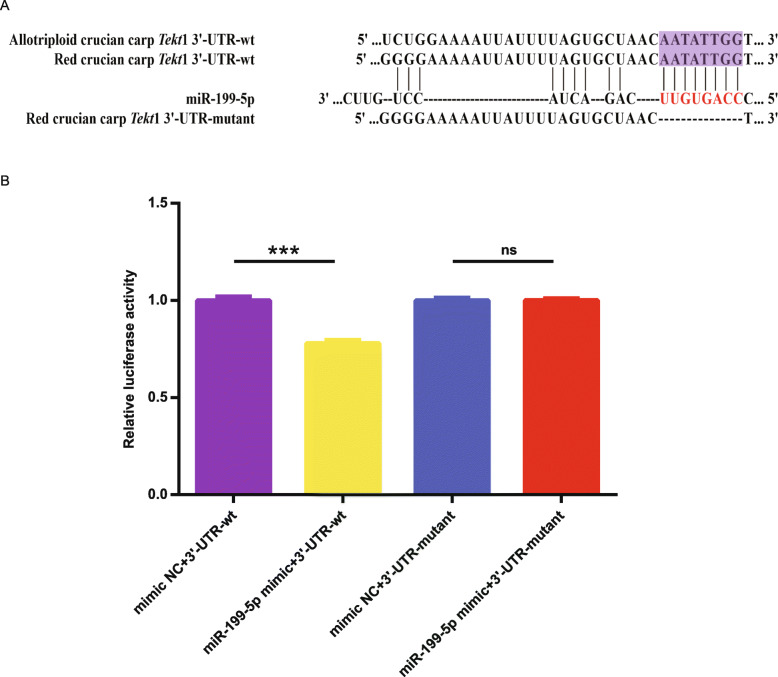
Validation of *Tekt*1 as a direct target of miR-199-5p. **A** Complementary sequences of miR-199-5p and the 3′-UTR region of the *Tekt*1. Red indicated the seed region of miR-199-5p; purple indicated the target 3′-UTR region of the *Tekt*1. **B** Validation of the interaction between miR-199-5p and the *Tekt*1 3′-UTR by a dual-luciferase reporter system. Luciferase reporters were linked with *Tekt*1 3′-UTRs containing either putative miR-199-5p-binding sites (3′-UTR-wt) or mutated miR-199-5p binding sites (3′-UTR-mutant). miR-199-5p mimic or mimic NC were cotransfected into HEK293T cells with luciferase-UTR constructs, and then luciferase activity was determined. The cells transfected with mimic NC plus 3′-UTR-wt were used to serve as the control group. Data were presented as mean ± S.E.M. (*n* = 6). Asterisk (*) indicated significant difference (***P < 0.001); ns indicated no significant difference

Based on our previous studies, the *Tekt*1 gene was selected as a potential target of miR-199-5p. Bioinformatic analysis predicted that the 278–285 nt position in the 3′-UTR region of *Tekt*1 gene was a complementary binding site for the 2–9 nt seed region of miR-199-5p (Fig. 3A).

To clarify the relationship between miR-199-5p and the *Tekt*1 gene, the constructed wild-type or mutant plasmids contained or deleted an 8-nt sequence in the seed region of miR-199-5p, targeting the 3′-UTR region of the *Tekt*1 gene, and miR-199-5p mimic or mimic NC were co-transfected into HEK293T cells (Fig. 3A). A luciferase assay showed that miR-199-5p significantly inhibited the relative luciferase activity of the wild-type *Tekt*1 reporter vector (*Tekt*1–3′-UTR-wt), whereas miR-199-5p did not alter the relative luciferase activity of the mutated *Tekt*1 reporter vector (*Tekt*1–3′-UTR-mutant) (Fig. 3B). Therefore, luciferase reporter assay unequivocally suggested that miR-199-5p negatively regulated *Tekt*1 expression by directly targeted binding to the putative binding site in the 3′-UTR.

### miR-199-5p is necessary for the spermiogenesis in vivo

**Fig. 4:**
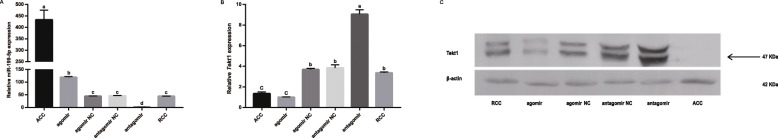
Expression profile of miR-199-5p and Tekt1 after intraperitoneal injection of agomir and antagomir. miR-199-5p agomir, miR-199-5p antagomir, agomir NC or antagomir NC was intraperitoneal-injected into diploids. The untreated groups and allotriploids were taken as control. The relative expression levels of **A** miR-199-5p and **B** *Tekt*1 were determined using qRT-PCR. **C** Western blot analysis. ACC, allotriploid crucian carp; RCC, diploid red crucian carp. Data were presented as mean ± S.E.M. (*n* = 3). The values with different lowercase letters differed significantly in expression level (*P* < 0.05)

Finally, we tested whether miR-199-5p was necessary for flagella formation to regulate spermiogenesis in animal model. Gain-of-function and loss-of-function of miR-199-5p in the testis were carried out using intraperitoneal injection of miR-199-5p agomir or miR-199-5p antagomir, respectively. The expression levels of miR-199-5p and *Tekt*1 were assessed by qRT-PCR, and the protein level of Tekt1 was evaluated by Western blot. Compared with the untreated groups and antagomir NC groups, the expression of miR-199-5p was obviously reduced in miR-199-5p antagomir-treated testis, and the expression of Tekt1 mRNA and protein was obviously upregulated. However, compared with the untreated groups and agomir NC groups, the expression of miR-199-5p was significantly elevated in miR-199-5p agomir-treated testis (Fig. 4A), and the expression of Tekt1 mRNA and protein was significantly downregulated (Fig. 4B, C). Meanwhile, the expression of miR-199-5p was evidently elevated in allotriploids (Fig. 4A), and the expression of Tekt1 mRNA and protein was evidently decreased (Fig. 4B, C). These results showed that miR-199-5p had a negative regulatory relationship with *Tekt*1.

**Fig. 5:**
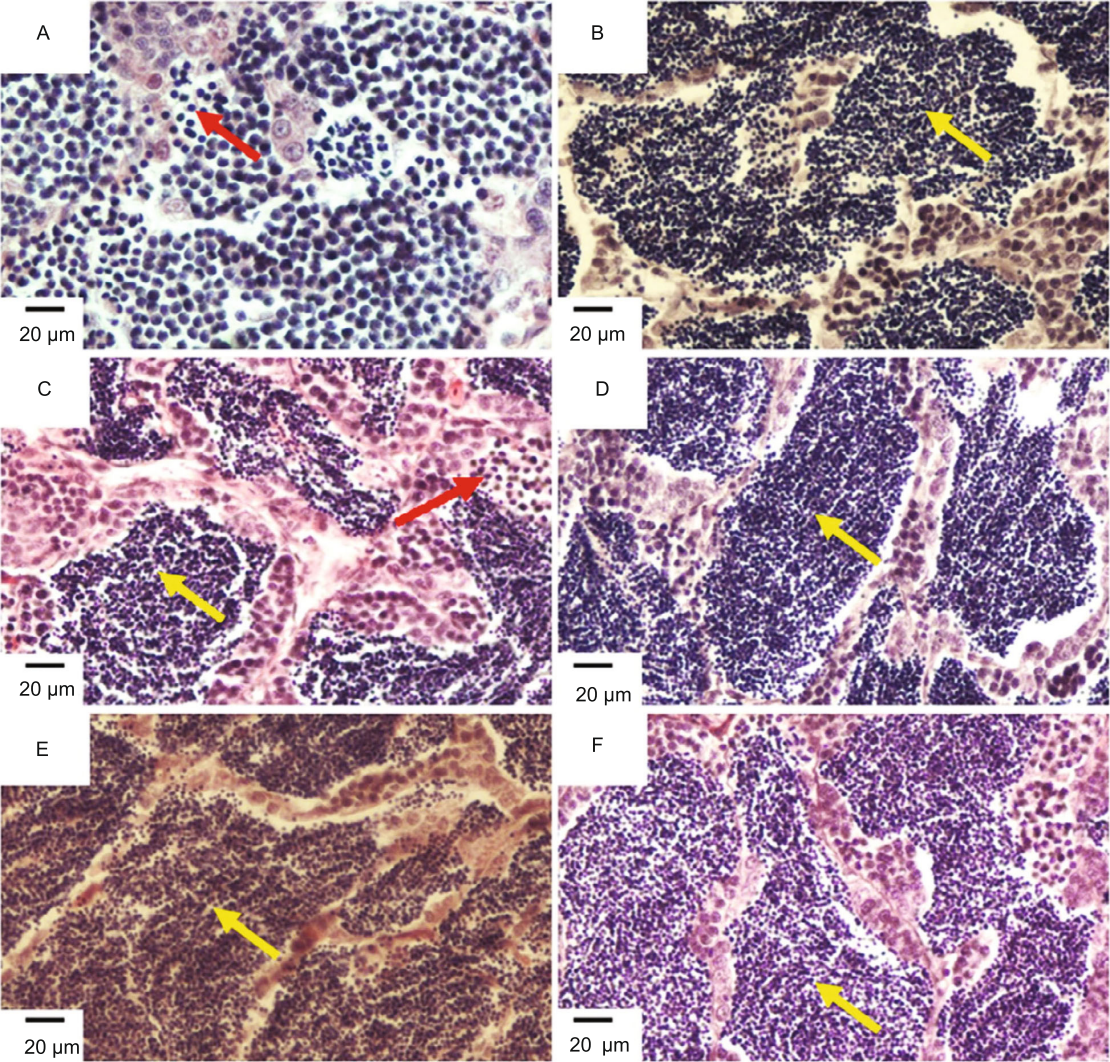
The testis microstructure of different-ploidy cyprinid fish. **A** The allotriploids with numerous apoptotic spermatids. **B** The untreated groups with mature spermatozoa. **C** The agomir intraperitoneal-injected diploids with a small number of apoptotic spermatids. **D** The agomir NC intraperitoneal-injected diploids with mature spermatozoa. **E** The antagomir intraperitoneal-injected diploids with mature spermatozoa. **F** The antagomir NC intraperitoneal-injected diploids with mature spermatozoa. Red arrow, apoptotic spermatids; yellow arrow, spermatozoa. Bar = 20 μm

**Fig. 6:**
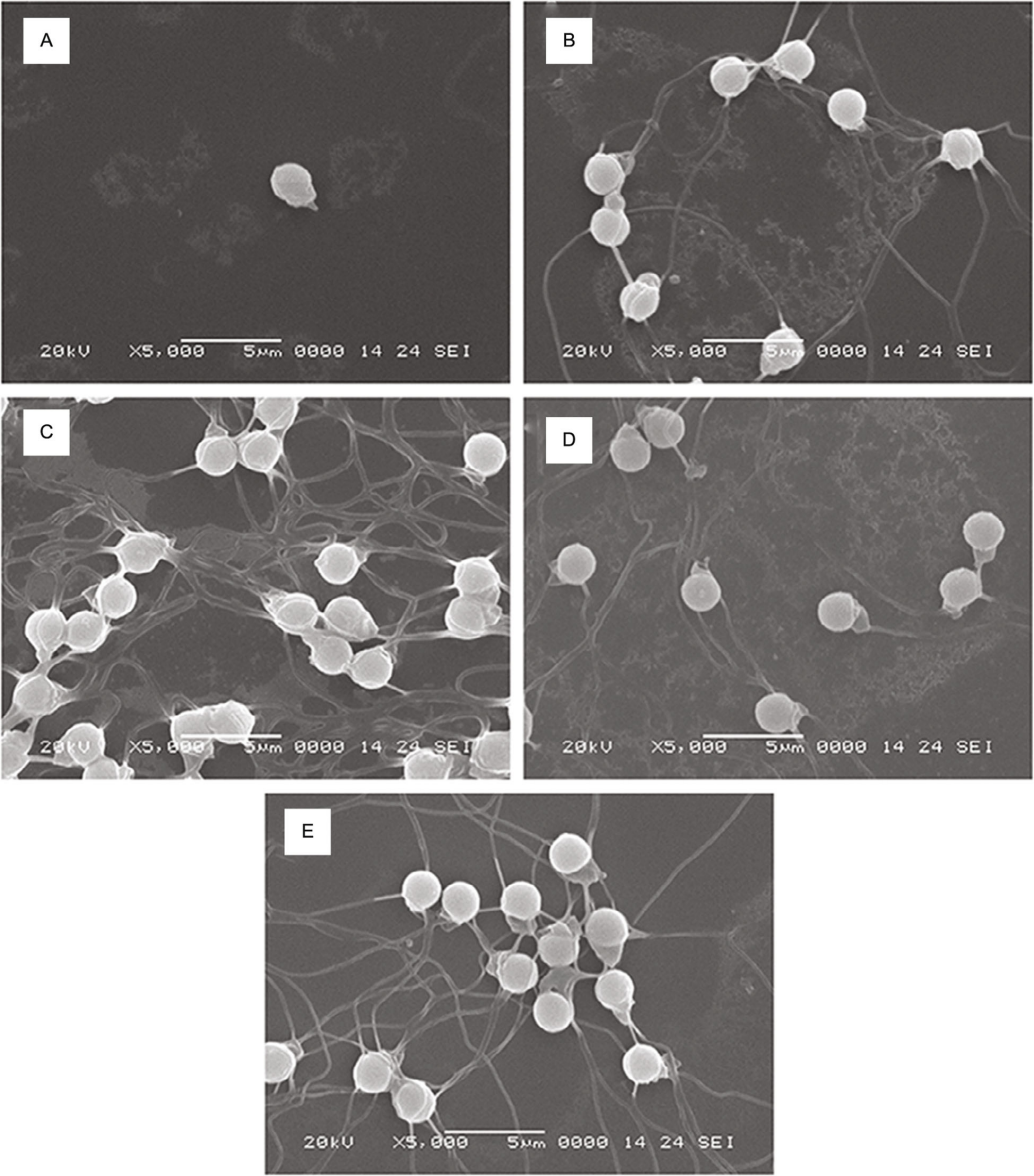
Spermatozoa smear scanning electron microscopy. **A** The agomir intraperitoneal-injected diploids spermatozoa. **B** The agomir intraperitoneal-injected diploid fish spermatozoa. **C** The antagomir intraperitoneal-injected diploids spermatozoa. **D** The antagomir NC intraperitoneal-injected diploids spermatozoa. **E** The untreated diploid fish spermatozoa. Bar = 5 μm

**Fig. 7:**
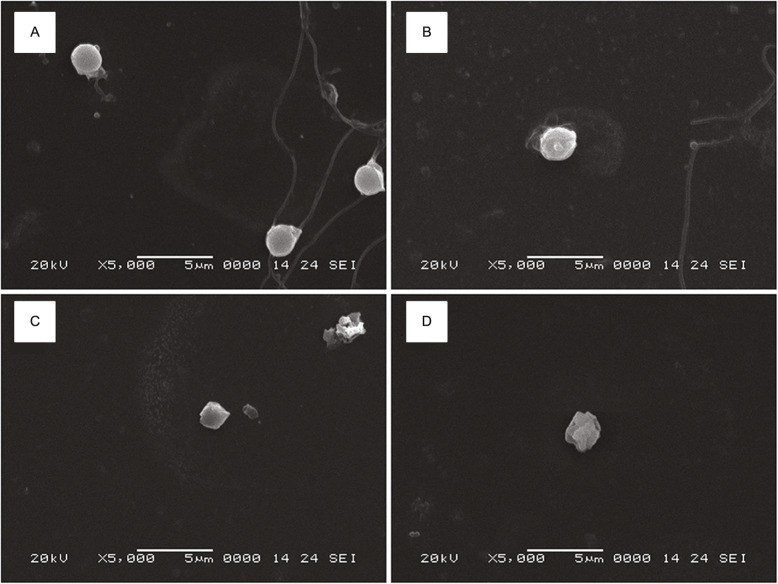
Testis tissue scanning electron microscopy. **A**, **B** The agomir intraperitoneal-injected diploids testis. **C**, **D** The allotriploids testis. Bar = 5 μm

Histological analysis of the testis of diploids and allotriploids was observed using H&E Staining. Cell apoptosis of some spermatids in the seminiferous lobules of allotriploids was found (Fig. 5A). The lobule lumens of the untreated groups, agomir NC groups and antagomir NC groups were full of spermatozoa, and there were a large number of spermatocytes on the spermatocyst wall (Fig. 5B, D, F). The lobule lumens of the antagomir groups were full of spermatozoa, and there were a few spermatocytes on the spermatocyst wall (Fig. 5E). There were a lot of spermatozoa in the lobule lumens of agomir groups, but cell apoptosis of some spermatids was found (Fig. 5C). We speculated that agomir-treated method might cause partial disruption of spermiogenesis. Besides, we further investigated the sperm morphology of diploids and allotriploids by scanning electron microscopy. The results showed that the sperm morphology of agomir NC groups, antagomir groups and antagomir groups was similar to that of untreated groups, mainly composed of head, midpiece and flagella, and the head was round (Fig. 6B-E). However, the sperm morphology of agomir groups and allotriploids did not have flagella structure, and the cytoplasmic membrane surface of some sperm heads was not smooth (Fig. 6A; Fig. 7).

## Discussion

The testis is a significant reproductive organ in male animals, and spermatogenesis exerts a crucial role in the reproduction of animals, including fish. Normal spermatozoa are of vital importance for the generation of offspring and species evolution. So far, studies on the biology of teleost fish spermatozoa have mainly concentrated on spermatozoon ultrastructure, sperm motility and sperm cryopreservation [43–48]. The sperm flagella are mainly composed of flagella axoneme, peripheral dense fibers and mitochondrial sheath, which is the main component of spermatozoa. The abnormal structure and function of sperm flagella will lead to sperm dyskinesia and male sterility. Therefore, the orderly flagella formation is a crucial stage for the formation of normal spermatozoa.

On the basis of obtaining testis transcriptomes of allotriploids and diploid crucian carp, the genes associated with reproduction, development, cell movement and sperm flagella were differently expressed in allotriploids [9]. Therefore, we speculated that one of the main reasons for the sterility of male allotriploids was the change in the expression level of related genes during spermatogenesis. However, the sterility of male allotriploids was mainly due to the failure of spermatids to form normal mature spermatozoa during spermatogenesis [9]. Previous studies have shown that the protein encoded by *Tekt*1 plays an important role in the formation or motility of sperm flagella [24]. In different-ploidy crucian carp, *Tekt*1 expression in the telencephala, hypothalami, pituitaries, ovaries, testes, kidneys, livers, hearts, spleens, skins and muscles was analyzed by RT-qPCR. The results showed that *Tekt*1 mRNA was expressed in all detected tissues, but especially highly expressed in the testis, indicating that *Tekt*1 was mainly present in the testis. Besides, researchers have also found the presence of TEKTs in the mammalian brain, retina, and other tissues containing cilia cells [17]. It is speculated that *Tekt*1 may play a contributory role in spermatogenesis as well as other life activities. Significantly, the expression pattern of *Tekt*1 in testis of sterile allotriploids was lower than that of fertile diploids during the spermiation period, while the expression pattern of *Tekt*1 in testis of sterile allotriploids was higher than that of fertile diploids during the pre-spermiation period. During the breeding season, the spermatids in the seminiferous lobules of sterile allotriploids appeared vacuoles and degeneration, and no normal mature spermatozoa were observed. However, the seminiferous lobules of fertile diploids were full of mature spermatozoa [49, 50]. These results revealed that the *Tekt*1 expression in fish testis was closely related to its development.

miRNAs have important implications for diverse pathophysiological processes such as cell proliferation, differentiation and apoptosis by regulating their mRNA targets [51–53]. Currently, the hotspots and difficulties in miRNA studies are the deeper mining of the target genes regulated by miRNA to further understand the mechanisms of their role in cells. It is estimated that approximately 60% of human protein-coding genes may be regulated by miRNAs [54]. The well-defined expression profiles of miRNAs have been exploited in clinical studies, and human spermatozoal or seminal plasma miRNAs have been explored as potential biomarkers for male sterility [32]. In the male reproductive system, miRNAs play central roles in various processes of spermatogenesis by strictly modulating the expression of their targets at the transcriptional and posttranscriptional level [55–58]. When the compacting sperm nucleus is transcriptionally inhibited, the posttranscriptional regulation becomes prominent during the late stages of spermatogenesis [59]. miRNAs are important regulators of gene expression, which mainly function posttranscriptionally to control the capabilities of their target mRNAs [59]. For example, has-miR-888 maintained sperm flagellar motility and mature sperm morphology by targeting *spag1* and *spag6* [60]. However, their functions in spermiogenesis have not been studied in detail. Since the role of miRNA in the regulation of *Tekt*1 expression in allotriploid fish is still unclear. Our study proposed for the first time that miR-199-5p regulated *Tekt*1 expression by binding to the 3′-UTR of *Tekt*1 mRNA in diploid red crucian carp and allotriploid crucian carp. Here, we focused our analysis on miR-199-5p and the results showed that miR-199-5p exerted a crucial role in spermiogenesis by targeting *Tekt*1. The differential expressions of miR-199-5p in different tissues of the same fish and different-ploidy crucian carp were also analyzed. The results showed that miR-199-5p was widely expressed in all detected tissues, indicating that miR-199-5p might be involved in the regulation of many life activities of the body. miR-199-5p is also expressed in various tissues such as the brain, liver, kidney, blood vessel, visceral smooth muscle, ovary, testis, myocardium and endothelial cells [61–65]. The expression trend of miR-199-5p during both the spermiation and pre-spermiation periods was exactly opposite to that of *Tekt*1, indicating that *Tekt*1 was a downstream effector that mediated the action of miR-199-5p. We provided evidence that miR-199-5p functioned as a direct negative regulator of spermatogenic *Tekt*1. miR-199-5p can directly repress Tekt1 protein expression through its binding to a specific binding site in the 3′-UTR region of the *Tekt*1, thereby negatively regulating spermiogenesis.

Besides, experimental methods were used to further study the changes in miRNA target gene expression after regulating a single miRNA level. The role of most studied miRNAs is based on the in vitro studies of miRNA small molecular agents for knockdown or overexpression [66–68]. Research on miRNA-based molecular drug design is currently mainly focused on the design of small molecules with miRNA as the target to antagonize its effect on target genes (such as antagomirs) or mimic miRNA to enhance its effect on target genes (such as agomirs). In this study, antagomir and agomir were utilized to knockdown and overexpress miR-199-5p in vivo, respectively. Antagomirs are chemically modified, cholesterol-conjugated single-stranded RNA molecules, which are complementary to mature target miRNAs. They can specifically and effectively inhibit endogenous miRNA expression [69]. Agomirs are specially labeled chemically modified double-stranded miRNA. They can regulate the biological function of the target gene by mimicking endogenous miRNA. In this study, we found that miR-199-5p overexpression by agomir significantly downregulated *Tekt*1 expression, which lead to spermatids apoptosis and sperm tail defects. However, the silencing of miR-199-5p by antagomir exhibited perfectly opposite effects. Downregulation of *Tekt*1 expression and failure of spermatogenesis in miR-199-5p agomir intraperitoneal-injected testis proved that the ‘miR-199-5p-*Tekt*1’ pathway exerts a crucial role in supporting spermiogenesis.

## Conclusions

The present study provided novel data that miR-199-5p negatively regulated the expression of *Tekt*1 in the testis. These results are of paramount significance to understand the regulatory mechanism of spermiogenesis and the sterility mechanism of male allotriploid crucian carp.

## Data Availability

All the datasets used and/or analysed throughout the present study are available from the corresponding author on reasonable request.

## Abbreviations

3′-UTRs: 3′-untranslated regions
ANOVA: Analysis of variance
DMEM: Dulbecco’s modified Eagle’s medium
FBS: Fetal bovine serum
H&E: Hematoxylin and eosin
HBSS: Hank’s Balanced Salt Solution
miRNAs: microRNAs
PBS: Phosphate-buffered saline
PLB: Passive lysis buffer
PVDF: Polyvinylidene difluoride
qRT-PCR: Quantitative real-time PCR
SDS-PAGE: Sodium dodecyl sulphate-polyacrylamide gel electrophoresis
TBS: Tris-buffered saline
TBST: TBS solution supplemented with Tween-20
TEKTs: Tektins
